# Development of a Multiplex-PCR probe system for the proper identification of *Klebsiella variicola*

**DOI:** 10.1186/s12866-015-0396-6

**Published:** 2015-03-13

**Authors:** Ulises Garza-Ramos, Jesús Silva-Sánchez, Esperanza Martínez-Romero, Perla Tinoco, Marisol Pina-Gonzales, Humberto Barrios, Jesús Martínez-Barnetche, Rosa Elena Gómez-Barreto, Juan Tellez-Sosa

**Affiliations:** Departamento de Diagnóstico Epidemiológico, Av. Universidad # 655, Col. Sta. Ma. Ahuacatitlán, C.P. 62100 Cuernavaca, Morelos Mexico; Centro de Ciencias Genómicas (CCG), Universidad Nacional Autónoma de México (UNAM), Cuernavaca, Morelos Mexico; Instituto Nacional de Salud Pública (INSP), Centro de Investigación Sobre Enfermedades Infecciosas (CISEI), Departamento de Inmunología, Cuernavaca, México

**Keywords:** Pathogen, Genome comparison, Prevalence, ESBL, Endophytic diazotrophic bacteria, Symbiosis

## Abstract

**Background:**

*Klebsiella variicola* was very recently described as a new bacterial species and is very closely related to *Klebsiella pneumoniae*; in fact, *K. variicola* isolates were first identified as *K. pneumoniae*. Therefore, it might be the case that some isolates, which were initially classified as *K. pneumoniae*, are actually *K. variicola*. The aim of this study was to devise a multiplex-PCR probe that can differentiate isolates from these sister species.

**Result:**

This work describes the development of a multiplex-PCR method to identify *K. variicola.* This development was based on sequencing a *K. variicola* clinical isolate (801) and comparing it to other *K. variicola* and *K. pneumoniae* genomes. The phylogenetic analysis showed that *K. variicola* isolates form a monophyletic group that is well differentiated from *K. pneumoniae*. Notably, the isolate *K. pneumoniae* 342 and *K. pneumoniae* KP5-1 might have been misclassified because in our analysis, both clustered with *K. variicola* isolates rather than with *K. pneumoniae*. The multiplex-PCR (M-PCR-1 to 3) probe system could identify *K. variicola* with high accuracy using the shared unique genes of *K. variicola* and *K. pneumoniae* genomes, respectively. M-PCR-1 was used to assay a collection of multidrug-resistant (503) and antimicrobial-sensitive (557) *K. pneumoniae* clinical isolates. We found *K. variicola* with a prevalence of 2.1% (23/1,060), of them a 56.5% (13/23) of the isolates were multidrug resistant, and 43.5% (10/23) of the isolates were antimicrobial sensitive. The phylogenetic analysis of *rpoB* of *K. variicola*-positive isolates identified by multiplex-PCR support the correct identification and differentiation of *K. variicola* from *K. pneumoniae* clinical isolates.

**Conclusions:**

This multiplex-PCR provides the means to reliably identify and genotype *K. variicola.* This tool could be very helpful for clinical, epidemiological, and population genetics studies of this species. A low but significant prevalence of *K. variicola* isolates was found, implying that misclassification had occurred previously. We believe that our multiplex-PCR assay could be of paramount importance to understand the population dynamics of *K. variicola* in both clinical and environmental settings.

**Electronic supplementary material:**

The online version of this article (doi:10.1186/s12866-015-0396-6) contains supplementary material, which is available to authorized users.

## Background

The genus *Klebsiella* belongs to the *Enterobacteriaceae* family and comprises Gram-negative opportunistic non-motile pathogens with a mucoid aspect. The species that comprise the genus *Klebsiella* are ubiquitous in nature and are present in three common habitats: i) the environment, where they can be found in water, soil and plants; ii) the mucous surfaces of mammals, [1] and iii) in symbiosis with insects [2]. In plants, they are endophytic and diazotrophic bacteria; in other words, they associate with plants by colonizing their internal tissues and providing enough nitrogen that the hosts mitigate their nitrogen deficiency [3]. In mammals, these bacteria colonize the upper respiratory tract and the gastrointestinal tract. Most *Klebsiella*-related infections are associated with hospitalization. These pathogens are the causative agents of 7 to 10% of all nosocomial infections reported in Europe, Latin-America and North America [1]. *K. variicola* was described as a new bacterial species in 2004 [4] based on the phylogenetic analysis of six housekeeping (*rpoB, gyrA, nifH, infB, phoE* and *mdh*) genes and DNA-DNA hybridizations. This work identified that 8% of the *K. pneumoniae* isolates corresponded to *K. variicola*, in both clinical isolates and environmental isolates [4]. Subsequently, multidrug-resistance phenotypes and extended-spectrum β-lactamase (ESBL) producers were identified for *K. variicola* during an intra-hospital outbreak at a pediatric hospital in Mexico [4,5]. Additionally, this species has been recently reported as a symbiotic nitrogen-fixing bacteria and has frequently been isolated from the fungus gardens of leaf-cutter ant colonies collected in Central and South America [2]. To date, the correct identification of *Klebsiella* species has not been easily achieved in microbiological laboratories because several species of this genus share similar biochemical profiles. Commercial kits, both manual and automated, do not include some of the organisms in their databases or the necessary substrates to differentiate species [6]. Some studies have been expressly designed to identify biochemical markers that can differentiate *K. variicola* from *K. pneumoniae*. For instance, the inability to ferment adonitol, which was one of the characteristics used by Rosenblueth *et al.* [4]. A recent study rejected this proposal because it was determined that this biochemical characteristic is variable among different isolates. Currently, the differentiation of *K. variicola* and *K. pneumoniae* using biochemical tests is not possible [7]. To date, *K. variicola* is one of the species of the genus *Klebsiella* that is not even included in commercial kits, and it has been described in several environments as a pathogen in humans (causing nosocomial infections), a symbiont in insects [2] and an endophyte in plants [4]. This lack of microbiology and molecular tools to differentiate these bacterial species may be reflected in misidentification, such as the case of *K. pneumoniae* 342, an endophyte in plants [3], which has been recently included within the cluster of the *K. variicola* species [8,9].

We describe the development of a multiplex-PCR method to identify *K. variicola;* notably, this development was based on sequencing a *K. variicola* clinical isolate (801) and comparing it to other *K. variicola* and *K. pneumoniae* genomes. To complement this, we conducted an array of phylogenetic analyses of selected loci that clearly differentiate *K. variicola* from *K. pneumoniae*. Furthermore, we describe the prevalence and characteristics of *K. variicola* among *K. pneumoniae* isolates (both multidrug resistant and sensitive) obtained from Mexican hospitals.

## Methods

### *K. variicola* 801 clinical isolate and genome sequencing

The *K. variicola* 801 isolate was obtained from the blood of a newborn with pneumonia at the Children’s Hospital in Tabasco, Mexico in 1996. This isolate, together with ten additional isolates (803–812), belongs to an intra-hospital outbreak identified at this hospital [5]. The *K. variicola* 801 clinical isolate was selected for genome sequencing based on the phylogenetic analysis of six housekeeping (*rpoB, gyrA, nifH, infB, phoE* and *mdh*) [4]. The genome was assembled and annotated using the bioinformatics software Maq (Mapping assembly) and MicroScope platform (https://www.genoscope.cns.fr/agc/microscope/home/index.php), respectively.

### Ethics statement

This project was exempt from review by the Ethic Commission at INSP because it does not involve human subjects and/or it is not an academic study and/or it does not include the analysis of data previously obtained from another study requiring the patients’ informed consent. On the other hand, the bacteria included in the study were obtained by routine procedures in each of the hospitals involved.

### Phylogenetic analyses of housekeeping genes

The nucleotide sequences of five housekeeping genes, *gyrA*, *phoE*, *infB, mdh* and *rpoB*, were obtained from *K. variicola* 801, *K. variicola* At-22, *K. pneumoniae* 342, *K. variicola* BZ19, *K. variicola* CAG:634, *K. pneumoniae* KP5-1, *K. pneumoniae* MGH78578, *K. pneumoniae* NTUH-K2044, *K. pneumoniae* KTCT 2242, *K. oxytoca* KTCT 1686, *Salmonella enterica* Ty21a, *Escherichia coli* K12 MG1655 genomes. Using these concatenate sequences of the five housekeeping genes, a maximum-likelihood phylogeny was generated using Mega v5.05 [10], with a Tamura-Nei model and 1,000 bootstrap replications.

### Comparison and functional category of shared unique proteins of *K. variicola* and *K. pneumoniae* genomes

The aim of this analysis was to identify the shared unique proteins of *K. variicola* (Kv_801, Kv_At-22 and Kp_342 [available at the moment of analysis]) and *K. pneumoniae* (Kp_NTUHK2044 and Kp_MGH78578) genomes. The predicted proteomes of *K. variicola* 801 (Kv_801) [5,246 proteins], *K. variicola* At-22 (Kv_At22) [5,057 proteins], *K. pneumoniae* 342 (Kp_342) [5,766 proteins], *K. pneumoniae* MGH78578 (Kp_MGH78578) [5,184 proteins] and NTUH-K2044 (Kp_NTUH-K2044) [5,262 proteins] were considered for comparisons. All of the comparisons were carried out using the BLASTp program with default values [11] (considers sequences as homologous if they have approximately 40% amino acid identity and 70% amino acid sequence coverage). For determining the shared unique proteins of *K. variicola*, we proceeded as follows: first, Kv_801 and Kv_At-22 were compared, and the set of shared proteins found in this comparison was then compared with Kp_342 to identify the shared proteins among the *K. variicola* genomes. Subsequently, the shared proteins among the *K. variicola* genomes were compared with the *K. pneumoniae* proteomes (Kp_MGH78578 and Kp_NTUH-K2044) to define the unique proteins sequences of *K. variicola*. A similar logic was used to define the proteins unique to *K. pneumoniae*; briefly, Kp_MGH78578 and Kp_NTUH-K2044 were compared, and the sequences shared by both were then compared against the *K. variicola* genomes (Kv_801, Kp_342 and Kv_At-22), thus identifying the shared unique protein sequences of *K. pneumoniae*. Next, we used the megablast option, which was designed for comparisons of highly similar sequences (percent identity ≥95%), to rule out that proteins unique to either *K. variicola* At-22 or *K. pneumoniae* 342 could be present in other related bacterial species. The functional category of the unique proteins identified in the *K. pneumoniae* and *K. variicola* genomes was determined using the BLASTp program [11]. (All non-redundant GenBank CDS translations + PDB + SwissProt + PIR + PRF excluding environmental samples from WGS projects). The proteins involved in the horizontal transfer of genetic material or those present in other organisms were eliminated. The proteins with a known or unknown (putative) function unique to *K. pneumoniae* and *K. variicola* were saved.

### Design of oligonucleotides, *mtnC* gene analysis and conditions for single- and multiplex-PCR

Oligonucleotides specific for shared unique genes identified in *K. variicola* and *K. pneumoniae* were designed using a similar melting temperature (mT), between 58°C and 62°C, and amplifying different amplicon lengths (from 340- to 888-bp). The analysis of the *mtnC* gene code for a protein specific to the genus *Klebsiella spp* [12,13] was used as a molecular marker of *Klebsiella* genera. Nucleotide analysis by BLASTn of the *mtnC* gene was carried out, and the alignment considered the gene sequences corresponding to *K. pneumoniae* NTUH-K2044 (GenBank ID: AP006725.1), *K. pneumoniae* MGH78578 (GenBank ID: CP000647.1), *K. pneumoniae* 342 (GenBank ID: CP000964.1), *K. variicola* At-22 (GenBank ID: CP001891.1) and *K. oxytoca* (GenBank ID: U00148.1). Multiple alignment from these sequences was performed using MEGA 5 [10]. Subsequently, by determining *in vitro* that the designed oligonucleotides are specific to *K. variicola, K. pneumoniae* and *Klebsiella* spp., the selected genes were amplified by single PCR using standard conditions. DNA from control strains such as *K. pneumoniae* ATCC 13883, *K. variicola* ATCC BAA-830 T (CFNE 2004 T), *R. terrigena* ATCC 33257, *R. planticola* ATCC 33531, and *K. oxytoca* ATCC 49134 were used. DNA was extracted using thermal shock, and by boiling and freezing at 96°C and 4°C, respectively. The *rpoB* gene was included as an amplification control for single PCR using the CM7 [4] and rpoB-M (5′GAGTCAACGGCAACAGCACG3′) oligonucleotides.

The conditions for the multiplex amplification of the shared genes unique to *K. variicola*, *K. pneumoniae* and the genus *Klebsiella* (*mtnC*) were determined. The following oligonucleotides and concentrations were proposed (Table 1): KV770-F and -R, KP888-F and -R and KmtnC-F and -R (named M-PCR-1), KV1615-F and -R, KP878-F and -R and KmtnC-F and -R (named M-PCR-2) (5 pmol/reaction of *K. variicola* and *Klebsiella spp*; 25 pmol/reaction of *K. pneumoniae*). In the combination of KV1000-F and -R, KP888-F and -R and KmtnC-F and -R (named M-PCR-3), the oligonucleotide concentrations were 5 pmol/reaction of *K. pneumoniae*, 1 pmol/reaction of *K. variicola,* and 0.2 pmol/reaction of the genus *Klebsiella*, respectively (Table 1). The amplified fragments underwent electrophoresis in a 1.2% agarose gel at 100 V for one hour in 1× TAE buffer solution (40 mM Tris–HCl, 2 mM acetic acid, 1 mM EDTA); the gel was dyed with ethidium bromide (5 μg/ml). The PCR products were purified using the High Pure PCR Product Purification kit (Roche) following the manufacturer’s recommendations. Thereafter, the amplified products were sequenced using the ABI PRISM 3100 system (Perkin-Elmer Division; Applied Biosystems), using the Sanger method with a Big-Dye Terminator Kit. The sequences were analyzed by BLASTn (http://blast.ncbi.nlm.nih.gov/Blast.cgi).

**Table 1:** **Amplification conditions, oligonucleotide combinations, sequence and amplification fragment of multiplex-PCR for** ***K. variicola*** **identification**

**Amplification conditions** ^**a**^	**Name of combination primers**	**Shared unique genes, oligonucleotides and sequence (5′- 3′) of each bacterial specie**					
		***K. pneumoniae***	**Amplification fragment (bp)**	***K. variicola***	**Amplification fragment (bp)**	***Klebsiella spp.***	**Amplification fragment (bp)**
		phosphohydrolase		phosphoglycerate mutase		phosphopentane phosphatase (*mtnC*)	
1	M-PCR-1	KP888-F: AAGCAAGCCAGAACAGAAAG	888	KV770-F: TCCCGAGGTTCACATTTCC	449	KmtnC-F: CCGCCGACCTTATCACTAC	340
		KP888-R: ACTTCGGTTTTATCCAGGTC		KV770-R: AGCGGGTGAACGTCGATAC		KmtnC-R: AGCGGGTGAACGTCGATAC	
		transferase (*yphG*)		N-acetyltransferase		phosphopentane phosphatase (*mtnC*)	
1	M-PCR-2	KP878-F: ACCGATAACCAGCCTGACTT	878	KV1615-F: ACACAACATTTCAGGCGGCT	499	KmtnC-F: CCGCCGACCTTATCACTAC	340
		KP878-R: CTTTCTTCTGCCCACTGTTG		KV1615-R: GGGCGTGGCTTTTTTCATCG		KmtnC-R: AGCGGGTGAACGTCGATAC	
		phosphohydrolase		thiopurine S-methyltransferase		phosphopentane phosphatase (*mtnC*)	
2	M-PCR-3	KP888-F: AAGCAAGCCAGAACAGAAAG	888	KV1000-F: CTGGGATGTGGCAATGGTG	438	KmtnC-F: CCGCCGACCTTATCACTAC	340
		KP888-R: ACTTCGGTTTTATCCAGGTC		KV1000-F: AAACTGCGCCTGCTGTATC		KmtnC-R: AGCGGGTGAACGTCGATAC	

^a^Multiplex-PCR conditions used under the oligonucleotides combinations. 1: 5pmol/reaction of *K. variicola* and *Klebsiella spp*, 25pmol/reaction of *K. pneumoniae*; 2: 25 pmol/reaction of *K. pneumoniae*, 5 pmol/reaction of *K. variicola* and 1 pmol/reaction of *Klebsiella spp.*

### Screening of *K. variicola* among *K. pneumoniae* clinical isolates

Using the multiplex-PCR development in the present work (Table 1), *K. variicola* was screened using M-PCR-1 (Table 1) among 1,331 clinical isolates previously identified as *K. pneumoniae.* The isolates were obtained from 28 different hospitals in Mexico (including pediatric and general hospitals) and included a period between 1990 and 2013 (Additional file 1). For the study, 1,060 isolates were included (according to the PFGE pattern and hospital origin); they comprised 79.6% of the total and were identified using the API 20E system (Clinical Diagnostic, BioMérieux). In this strain collection, 47.4% (503) corresponded to multidrug-resistant and ESBL-producing isolates, and 52.6% (557) corresponded to isolates that were susceptible to most antibiotics (non-ESBL-producer). The latter isolates corresponded only to two hospitals (Additional file 1). All of the isolates obtained from the *K. variicola* outbreak at a pediatric hospital in Tabasco [5] and the *K. variicola* endophytic isolates obtained from maize (n = 3), sugar cane stem (T29A), rice roots (CFNE 2006), banana soot, leaves and stem (F2R9 (ATCC BAA-830 T), 6A2 and VI, respectively were included in the study. The latter isolate was previously isolated and characterized by Rosenblueth *et al.* [4]. The *K. variicola*-positive isolates were confirmed using M-PCR-2 and M-PCR-3. Initially, the DNA of three clinical isolates was mixed, and the mixtures that were *K. variicola*-positive were assayed subsequently by individual multiplex-PCR.

### Phylogenetic analysis and characterization of *K. variicola*-positive isolates

The clinical and environmental isolates identified as *K. variicola* (22/30) and 21 *K. pneumoniae* clinical isolates were selected for the PCR amplification of *rpoB* genes, using the oligonucleotides from *K. pneumoniae* MLST [14]. Regarding *K. pneumoniae*, isolates were selected from different hospitals (Additional file 1). The PCR products were purified using the High Pure PCR Product Purification kit (Roche) following the manufacturer’s recommendations. Thereafter, the amplified products were sequenced in the ABI PRISMA 3100 system (Perkin-Elmer Division; Applied Biosystems) using the Sanger method with the Big-Dye Terminator Kit. The single-gene comparison for the *rpoB* DNA sequence was performed using Mega v5.05 [10]. The *rpoB* partial nucleotide sequence (501 bases) phylogeny was constructed using the maximum-likelihood method with a Tamura-Nei-parameter model and 100 bootstrap replications. Additionally, the chromosomal β-lactamase gene from the *K. variicola* 801 isolate was analyzed against the chromosomal encoded β-lactamases from *K. variicola* At-22, *K. variicola* CAG:634, *K. variicola* BZ19, *K. pneumoniae* 342 and *K. pneumoniae* KP5-1, *K. pneumoniae* MGH78578 and *K. pneumoniae* NTUH-K2044 isolates. LEN-type amino acid sequences were obtained from Pasteur Institute Home page (http://bigsdb.web.pasteur.fr/perl/bigsdb/bigsdb.pl?db=pubmlst_klebsiella_seqdef_public&page=downloadAlleles) and were included in the analysis from the 17- to 278-amino acid sequence (LEN-6 and LEN-14 alleles were excluded due to the partial sequence). The single-gene comparison for the LEN-alleles amino acid sequence was performed using Mega v5.05 [10]. The phylogeny was constructed using the maximum-likelihood method with a Jones-Taylor-Thornton-parameter model and 100 bootstrap replications.

In all *K variicola* isolates, the antimicrobial susceptibility against amoxicillin, AMX; cephalothin, CF; cefoxitin, FOX; cefuroxime, CXM; piperaciline, PIP; piperaciline/tazobactam, TZP; aztreonam, ATM; cefotaxime, CTX; ceftazidime, CAZ, gentamicin, GM; amikacin, AMK; ticarcillin, TIC; tetracycline, TET; nalidixic Acid, NAL; ciprofloxacine, CIP; levofloxacin, LUX and ESBL-production, respectively, were determined by disk diffusion and the combination disk method, with ceftazidime and cefotaxime applied individually and in combination with clavulanic acid, following the recommendations of the Clinical and Laboratory Standards Institute (CLSI) (M100-S21) [15]. In the first case, the results were interpreted as resistant R, intermediate I and susceptible S, according to the CLSI performance standard M100-S21.[15] *E. coli* ATCC 25922 was used as a reference strain for susceptibility testing. The CTX-M- and SHV-type ESBL genes were screened by PCR using specific primers [16]. The relationship of *K. variicola* isolates was determined by pulse-field gel electrophoresis (PFGE) [17] and interpreted by GelCompar II software (Applied Math, Kortrijk, Belgium). The similarity percentage was represented using a dendrogram derived from UPGMA and Dice coefficients (the band position tolerance and optimization were set at 1.0% and 1.0%, respectively). In addition, KPC-type-producing *K. pneumoniae* clinical isolates that had previously been described in Mexico [18,19] were included in the fingerprinting analysis.

## Results and Discussion

### *K. variicola* genome characteristics

The *K. variicola* 801 clinical isolate was pyrosequenced on a 454 Roche FLX TITANIUM platform; *de novo* assembly was conducted, revealing 91 contigs with an N50 of 131,409 pb; the estimated genome size is 5,790,474 bp with 15× coverage, and 99.82% of the bp were above Q40. Genome annotation identified a total of number of genomic objects (CDS, fCDS, rRNA, tRNA, miscRNA) of 5,736 and a number of CDS (total) of 5,620. The annotated genome sequence was deposited in the European Nucleotide Archive under accession number: CDMV01000001. In the present work, the genome of this *K. variicola* 801 clinical isolate was compared with another *K. variicola* and *K. pneumoniae* genomes. Based on those comparisons, a phylogenetic and comparative genomic analysis developed a multiplex-PCR probe system for the proper identification of *K. variicola.* Next, we used this method to evaluate the prevalence of *K. variicola* in a collection of isolates that had previously been identified by biochemical tests as *K. pneumoniae*.

### Phylogenetic analysis of *K. variicola* and *K. pneumoniae* isolates

**Figure 1:**
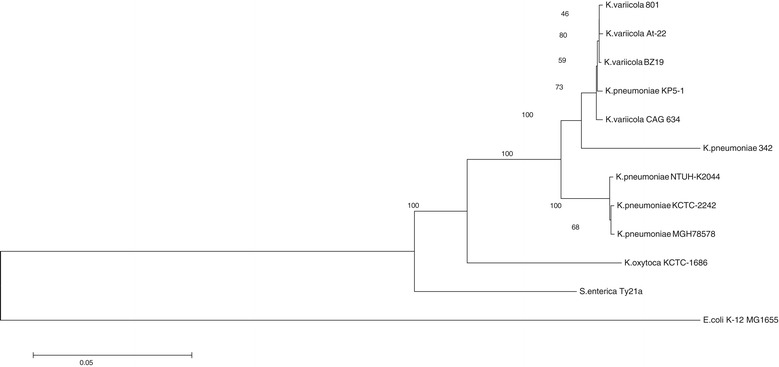
**The maximum-likelihood gene phylogenies of** ***K. variicola*** **and** ***K. pneumoniae*** **.** Bootstrapping of the gene tree was implemented to evaluate the support of the *K. variicola* and *K. pneumoniae* groups. The numbers next to the nodes are the bootstrap values, and the length of the branches has no meaning.

*K. variicola* and *K. pneumoniae* isolates, either environmental or clinical, have very similar phenotypic and biochemical characteristics. Thus, their differentiation through conventional or automated biochemical methods is not currently possible. Misclassification of *Klebsiella spp*. isolates could occur. With this view, a phylogenetic analysis of housekeeping genes was carried out from *K. variicola* and *K. pneumoniae* isolates. Figure 1 shows that *K. variicola* isolates and *K. pneumoniae* 342 clustered together to the exclusion of all of the *K. pneumoniae* isolates. There is full support, with a bootstrap value of 1,000 for the group composed of the *K. variicola* isolates and *K. pneumoniae* 342. The *K. pneumoniae* 342 endophytic strain clearly is a strain of *K. variicola*; this assumption was hypothesized when *K. variicola* was described as a new bacterial species [4] and was recently confirmed in other studies [9,20,21]. Thus, in the present study, this bacterium was considered to belong to the *K. variicola* species for the rest of the analysis.

In the seminal work that describes *K. variicola,* one of the defining characteristics of this species is its ability to fix nitrogen. Considering this characteristic, we checked for the presence of the *nifH* gene, which is essential for nitrogen fixation. The *nifH* gene was not identified in *K. pneumoniae* genomes (MGH78578, NTUH-K2004, KTCT 2242 and JM45). Nevertheless, BLASTn analysis of the *nifH* gene showed a match with different bacterial genera: *K. variicola* At-22, *K. variicola* CAG:634, *K. variicola* BZ19, *K. pneumoniae* 342, *K. pneumoniae* KP5-1, *K. pneumoniae* (X13303.1 and J01740.1), *K. oxytoca* (KTCT 1686), *Pantoea sp.* (At-9b), *Enterobacter sp.* (R4-368), *R. aquatilis* ATCC33071 and *P. stutzeri* (DSM4166) (data not showed). *nifH* genes belong to operon *nif*, which contains a 20-*nif* gene cluster involved in nitrogen fixation [22]. Historically, the *nif* gene cluster has been extensively characterized in *K. pneumoniae* [23]. However, Hazen *et al.* (2014) described the absence of the *nif* gene cluster in eight *K. pneumoniae* NTUH-K2044, MGH 78578, 1162281, JH1, MS 92–3, 1191100241, ATCC13884 and KCTC 2242 clinical isolates [8]. The ability of nitrogen fixation of the *K. variicola* 801 clinical isolate was described by Rosenblueth *et al.* (2004) [4]. Here, we confirm that this *K. variicola* clinical isolate contains *nifJ*-*nifQ* cluster genes (20 genes), with a >98 nucleotide identity with *K. variicola* At-22 and *K. pneumoniae* 342 *nif* cluster genes (data not showed). This analysis showed that the *nifH* gene is a clear characteristic of *K. variicola* isolates, and *K. pneumoniae* isolates that contain the *nifH* gene correspond rather to *K. variicola* species. As in the case of *K. pneumoniae* KP5-1 (CP008700), what was recently submitted to GenBank (04-JUN-2014) (see below).

A maximum likelihood phylogeny of LEN-type β-lactamase, chromosomal from *K. variicola* 801, *K. variicola* At-22, *K. variicola* CAG:634, *K. variicola* BZ19, *K. pneumoniae* 342 and *K. pneumoniae* KP5-1 genomes was constructed (Additional file 2). The *K. variicola* 801 isolates showed a new LEN-type allele, LEN-32 with one new amino acid change in the position 68 (S → M), which was deposited in the PasteurMLST data base (http://bigsdb.web.pasteur.fr/perl/bigsdb/bigsdb.pl?db=pubmlst_klebsiella_seqdef_public&page=downloadAlleles). LEN-13 was identified in *K. variicola* At-22 and *K. variicola* BZ19. LEN-10, LEN-16 and LEN-28 alleles were identified in *K. pneumoniae* CAG:634, *K. pneumoniae* KP5-1 and *K. pneumoniae* 342, respectively. Duplication in tandem of the LEN-28 gene with 14 other chromosomal genes (*lysR*-Deor) is contained in the *K. pneumoniae* 342 chromosome (data not shown). In the case of the *K. pneumoniae* MGH78578 and *K. pneumoniae* NTUH-K2004, the genomes have a different chromosomal β-lactamase, namely SHV-11, which is closely related to the LEN-type (Additional file 2). These chromosomally encoded β-lactamases are characteristic of *K. variicola* and *K. pneumoniae* species. They could not be used as molecular markers because the SHV-type β-lactamases have been broadly described for conjugative and non-conjugative plasmids in both *K. variicola* and *K. pneumoniae* isolates [5]. The LEN-type β-lactamases have not been described on plasmids. Furthermore, LEN-26 is chromosomally encoded in *Klebsiella* sp. 10982, which is a bacterium recently described as a phylogenetic and metabolic intermediate between the *K. variicola* endophyte and *K. pneumoniae* clinical isolates [8].

### Comparative analysis of the *K. variicola* and *K. pneumoniae* genomes

Very little is known concerning *K. variicola* or the role that it plays in the environment and hospitals. *K. variicola* was identified using molecular strategies based on phylogenetic analyses of housekeeping genes [4]. However, previously, analyzing *K. pneumoniae* clinical samples, Brisse and collaborators carried out phylogenetic analyses of *gyrA* and *parC* genes and the adonitol fermentation test [24]. They identified three different phylogenetic groups called KpI, KpII and KpIII with KpI being the largest. Group KpIII very likely belongs to *K. variicola* because it has *gyrA* sequences that are very similar to those found in *K. variicola* [4]. It is noteworthy that although there are differences between these groups, the phenotypic characteristics of *K. pneumoniae* do not change; it is only possible to differentiate these groups using gene sequencing. Comparative genome studies on *Klebsiella spp*. have been carried out [3,9,13], and the development of biochemical techniques to differentiate *K. variicola* from *K. pneumoniae* has also been tried. For example, Alves *et al.* [7] tried to implement a biochemical test that includes the adonitol-as-a-carbon-source test as a potential strategy to identify *K. variicola* among *K. pneumoniae* isolates. The adonitol test was found to be variable in both species and, therefore, not reliable to discriminate between these species. To date, a biochemical test to identify *K. variicola* has not yet been determined. Nonetheless, a study carried out by Van-Veen *et al*. [25] reported the identification of bacterial genus and species using mass spectrometry. The study comprised non-fermentative Gram-negative microorganisms, yeast and *Enterobacteriaceae*, including *K. pneumoniae* and *K. variicola*. This tool showed the correct identification of the *Klebsiella* genus, but an incorrect identification of species (minor error) was revealed regarding *K. variicola* in *K. pneumoniae*. Although this molecular technique is highly specific, it still has an error margin and thus cannot be used to identify *K. variicola*. The problems in identifying species within a bacterial genus are not specific to *K. pneumoniae* and *K. variicola*. Efforts to optimize the identification methods of highly related species of other microorganisms have also been tried out. Some tests that have been developed to identify species rely on molecular tools, as in the case of the genus *Salmonella* [26,27]. A different approach was addressed in this work, with a comparative analysis of the *K. variicola* and *K. pneumoniae* genomes (see methods). This comparison revealed 114 proteins shared by the *K. variicola* genomes but not present in *K. pneumoniae*. In the case of *K. pneumoniae* genomes, we identified 54 proteins that were shared but not present in *K. variicola* (Additional file 3). Excluding the genes involved in horizontal transfer, we identified 79 proteins shared by *K. variicola* and 40 proteins shared by *K. pneumoniae*. Of the proteins that were found to be unique to each species, those involved in metabolism and cellular structure were selected. Therefore, 20 metabolic proteins and 12 structural proteins were selected for *K. variicola*, and four metabolic proteins and six structural ones were selected for *K. pneumoniae* (Additional file 4). Next, we ruled out the gene sequences of the shared proteins unique to either *K. variicola* At-22 or *K. pneumoniae* 342 that are present in other related bacterial species (see methods). Therefore, the sequences selected were those exclusively present in the *K. variicola* At-22 and *K. pneumoniae* 342 genomes or the *K. pneumoniae* MGH78578 and NTUH-K2044 genomes. These were the three shared metabolic genes unique to *K. variicola* and two shared metabolic genes unique to *K. pneumoniae*. The specific oligonucleotides for the PCR amplification of shared unique genes were designed (Table 1).

### Characteristics of multiplex-PCR for *K. variicola* identification

The inclusion of a PCR amplification control that was also a gene characteristic of *Klebsiella* genera was considered, and the *mtnC* gene was selected. The phosphopentane phosphatase (*mtnC* gene) protein is part of the methionine salvage pathway, which is frequent in bacteria [12]. A recent study reported that the *mtnC* gene is only contained in *K. pneumoniae* [13]. We carried out a BLASTn (considering the Nucleotide collection [nr/nt] from NCBI database) search using as a query the nucleotide sequence of the *mtnC* gene taken from the genome of *K. pneumoniae* NTUH-K2044. The analysis revealed 22 similar gene sequences in the GenBank, seven of which were bacteria of the genus *Klebsiella* and showed an identity >95%. The closest match after those was the sequence of the *mtnC* gene corresponding to *Pantoea vagans* (49%). The *mtnC* gene from the *K. pneumoniae* NTUH-K2044, *K. pneumoniae* MGH78578, *K. pneumoniae* 342 and *K. variicola* At-22 genomes showed a 100% identity, followed by a 98% and a 96% identity from the *Klebsiella oxytoca* and *Enterobacter cloacae* ATCC 13047 and *Enterobacter* sp. 638 genomes, respectively. The design of the oligonucleotides of the *mtnC* gene from the genus *Klebsiella* was determined by selecting a fragment in which the sequences of *K. pneumoniae* NTUH-K2044, *K. pneumoniae* MGH78578, *K. pneumoniae* 342 and *K. variicola* At-22 isolates were identical. The oligonucleotides were named KmtnC-F and KmtnC-R and amplified a 340-bp fragment (Table 1).

**Figure 2:**
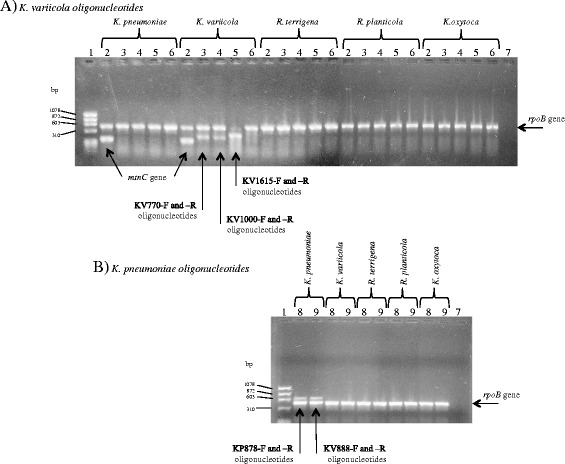
**Amplification by PCR of shared unique genes to** ***K. variicola*** **and** ***K. pneumoniae*** **. A)** Amplification of shared unique genes to *K. variicola* using the genomes of control strains such as *K. pneumoniae* ATCC 13883, *K. variicola* ATCC BAA-830 T, *R. terrigena* ATCC 33257, *R. platicola* ATCC 33531 and *K. oxytoca* ATCC 49134. **B)** Amplification of shared unique genes to *K. pneumoniae* using the genomes of control strains described above. Lane 1, ϕX174/Hae III; Lane 2, *mtnC* gene (KmtnC-F and -R oligonucleotides); Lane 3, phosphoglycerate mutase gene (KV770-F and -R oligonucleotides); Lane 4, thiopurine S-methyltransferase gene (KV1000-F and -R oligonucleotides); Lane 5, N-acetyltransferase gene (KV1615-F and -R oligonucleotides); Lanes 1 to 6, *rpoB* gene (CM7 and rpoB-M) in combination with oligonucleotides of shared unique genes *K. variicola*; Lane 7, *rpoB* oligonuclotides without DNA (CM7 and rpoB-M); Lane 8, Transferase gene (KP878-F and R oligonucleotides); Lane 9, phosphohydrolase gene (KP888-F and R oligonucleotides) (Table 1).

The oligonucleotides designed from the shared unique genes for *K. variicola* were as follows: KV770-F and KV770-R that amplify a 449-bp fragment of the gene encoding phosphoglycerate mutase protein; KV1000-F and KV1000-R that amplify a 438-bp fragment of the gene encoding thiopurine S-methyltransferase protein and KV1615-F and KV1615-R that amplify a 499-bp fragment of the gene encoding N-acetyltransferase protein (Table 1). Regarding the shared unique genes for the species *K. pneumoniae*, the following oligonucleotides were used: KP878-F and KP878-R that amplify an 878-bp fragment of the gene encoding transferase protein and KP888-F and KP888-R that amplify an 888-bp fragment of the gene encoding phosphohydrolase protein (Table 1). Both the *mtnC* gene and the shared unique genes of *K. variicola* and *K. pneumoniae* genomes, amplified by simple PCR, were highly specific to the respective bacterial genomes and *Klebsiella* genera (Figure 2A and B).

**Figure 3:**
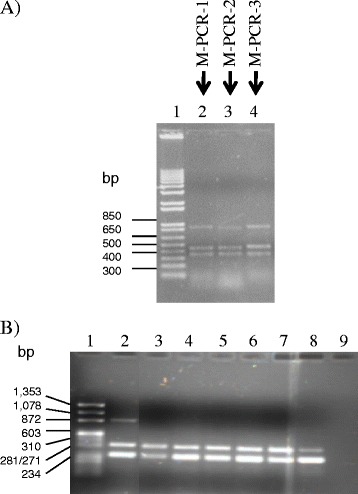
**Multiplex-PCR amplification of oligonucleotides combinations used for identification of** ***K. variicola*** **. A)** Lane 1, molecular weight 1 kb; Lane 2, oligonucleotide combination named M-PCR-1 (KV770-F/KV770-R, KP888-F/KP888-R and KmtnC-F/KmtnC-R); Lane 3, oligonucleotide combination named M-PCR-2 (KV1615-F/KV1615-R, KP878-F/KP878-R and KmtnC-F/KmtnC-R); Lane 4, oligonucleotide combination named M-PCR-3 (KV1000-F/KV1000-F, KP888-F/KP888-R and KmtnC-F/KmtnC-R) (Table 1). **B)** M-PCR-1 assayed on environmental endophytic *K. variicola* isolates. Lane 1, molecular weight ΦX174 DNA-Hae III; Lane 2, *K. pneumoniae* ATCC 13883 and *K. variicola* 801, DNA’s combination; Lane 3, *K. variicola* T29A; Lane 4, *K. variicola* 6A2; Lane 5, *K. variicola* CFNE 2006; Lane 6, *K. variicola* 3; Lane 7, *K. variicola* F2R9; Lane 8, *K. variicola* VI; Lane 9, negative control (without DNA).

After the single-PCR amplification, the conditions for multiplex-PCR were determined. A mixture of *K. variicola* 801 and *K. pneumoniae* ATCC 13883 DNA was used as the positive control. The proposed multiplex PCR assays were as follows (Table 1): oligonucleotides KV770-F and -R, KP888-F and -R and KmtnC-F and -R, named M-PCR-1; oligonucleotides KV1615-F and -R, KP878-F and -R and KmtnC-F and -R, named M-PCR-2 and the oligonucleotides combination of KV1000-F and -R, KP888-F and -R and KmtnC-F and -R, named M-PCR-3 (Figure 3A). Using the multiplex-PCR assays, all of the environmental endophytic *K. variicola* isolates [4] were identified correctly (Figure 3B). All of these experiments confirmed that the set of oligonucleotides of *K. variicola* and *K. pneumoniae* amplified only *K. variicola* and *K. pneumoniae* DNA, respectively.

### Prevalence, susceptibility and characteristics of *K. variicola* isolates

Finally, to evaluate our new multiplex PCR, we determined the prevalence of *K. variicola* isolates among a collection of *K. pneumoniae* isolates. The M-PCR-1 multiplex PCR assay was tested on a collection of 1,060 *K. pneumoniae* isolates (Additional file 1). The assay identified 23 *K. variicola* isolates, which corresponded to a prevalence of 2.1% (23/1,060)*.* The amount of *K. variicola* was smaller than that reported in previous studies; in 2004, two reports stated to have found *K. variicola* prevalences of 8% [4] and 11%, respectively [24]. Considering that the phylogenetic group KpIII found by Brisse *et al.* [24] corresponded to *K. variicola* isolates, as reported by Rosentblueth *et al.* [4]. Of the remaining 1,034 *K. variicola-*negative isolates corresponded to *K. pneumoniae* bacterial species. Of them, 21 *K. pneumoniae* clinical isolates were selected for the phylogenetic analysis of the *rpoB* gene (see below). Of the 23 (2.1%) *K. variicola-*positive isolates, 43.5% (10/23) were antimicrobial sensitive isolates. The antimicrobial susceptibility of these ten isolates was confirmed by disk diffusion against the main family of antimicrobials used in the hospital settings. These clinical isolates, together with the environmental *K. variicola* isolates, were sensitive to all of the antibiotics tested, except amoxicillin, with a negative-ESBL phenotype. Some clinical isolates showed an intermediate resistance to ticarcillin and nalidixic acid (Table 2). The multidrug-resistant *K. variicola* isolates showed a prevalence of 56.5% (13/23) and were ESBL producers (Table 2). These isolates showed resistance to amoxicillin, cephalothin, piperacillin, cefotaxime and ceftazidime, amikacin and ticarcillin. However, the isolates were sensitive to some β-lactam antibiotics (cefoxitin, cefuroxime and aztreonam) and to all quinolones and fluoroquinolones tested, as well as tetracycline (Table 2). With respect to ESBL genes, the ten isolates from the *K. variicola* outbreak identified in the hospital at Tabasco in 1996 showed SHV-2 [5] and SHV-2a ESBLs, with the latter being more prevalent (Table 2). In the other ESBL-producing *K. variicola* isolates, the ESBL SHV-5 and CTX-M-15 genes were identified, corresponding to isolates obtained at different hospitals, with multiple samples of origins and susceptibilities (Table 2).

**Table 2:** **Characteristics of endophytic and clinical** ***K. variicola*** **isolates**

**Isolate**	**Hospital** ^**a**^	**Isolation date**	**Origin of the sample**	**PFGE pattern**	**ESBL production**	**ESBL**	**Antimicrobial susceptibility** ^**b**^															
							**AMX**	**CF**	**FOX**	**CXM**	**PIP**	**TZP**	**ATM**	**CTX**	**CAZ**	**GM**	**AMK**	**TIC**	**TET**	**NAL**	**CIP**	**LUX**
1109	1	1996	Blood	NR	-	Negative	**R**	S	S	S	S	S	R	**R**	**R**	**R**	**R**	I	S	S	S	S
801	2	1996	Blood	A	+	SHV-2a	**R**	**R**	S	S	**R**	S	S	**R**	**R**	**R**	**R**	**R**	S	S	S	S
803	2	1996	Blood	A	+	SHV-2a	**R**	**R**	S	I	**R**	S	S	**R**	I	S	**R**	**R**	S	S	S	S
804	2	1996	Blood	A	+	SHV-2a	**R**	**R**	S	S	**R**	S	S	**R**	I	S	**R**	**R**	S	S	S	S
805	2	1996	Blood	A	+	SHV-2a	**R**	**R**	S	S	**R**	S	S	**R**	I	S	**R**	**R**	S	S	S	S
806	2	1996	Blood	A	+	SHV-2	**R**	**R**	S	S	**R**	S	S	**R**	I	S	**R**	**R**	S	S	S	S
807	2	1996	Blood	A	+	SHV-2a	**R**	**R**	S	S	**R**	S	S	**R**	**R**	S	**R**	**R**	S	S	S	S
808	2	1996	Blood	A	+	SHV-2a	**R**	**R**	**R**	S	**R**	S	S	**R**	I	I	**R**	**R**	S	S	S	S
809	2	1996	Blood	A	+	SHV-2a	**R**	**R**	**R**	S	**R**	S	I	**R**	**R**	**R**	**R**	**R**	S	S	S	S
811	2	1996	Blood	A	+	SHV-2a	**R**	**R**	**R**	S	**R**	S	S	**R**	**R**	I	**R**	**R**	S	S	S	S
812	2	1996	Blood	A	+	SHV-2a	**R**	**R**	**R**	S	**R**	S	S	**R**	**R**	I	**R**	**R**	S	S	S	S
1171	1	1998	Catheter	NR	-	Negative	**R**	S	S	S	S	S	S	S	S	S	S	I	S	S	S	S
1258	3	1999	Blood	NR	+	SHV-5	**R**	**R**	**R**	I	**R**	S	**R**	S	S	**R**	**R**	**R**	S	S	S	S
06-268	4	2007	Abscess	NR	+	CTX M- 15	**R**	**R**	S	S	S	S	**R**	**R**	**R**	S	S	I	S	S	S	S
8917	5	2011	sputum	NR	-	Negative	**R**	S	S	S	S	S	S	S	S	S	S	S	S	S	S	S
9635	6	2011	Catheter	NR	-	Negative	**R**	S	S	S	S	S	S	**R**	**R**	S	S	I	S	S	S	S
9351	6	2012	Secretion	NR	-	Negative	**R**	S	S	S	S	S	S	S	S	S	S	I	S	I	S	S
9352	6	2012	Secretion	NR	-	Negative	**R**	S	S	S	S	S	S	S	S	S	S	S	S	S	S	S
9353	6	2012	Biliary liquids	NR	-	Negative	**R**	S	S	S	S	S	S	S	S	S	S	I	S	S	S	S
4880	6	2013	Blood	NR	-	Negative	**R**	S	S	S	S	S	S	S	S	S	S	I	S	I	S	S
9326	6	2013	Secretion	NR	-	Negative	**R**	S	S	S	S	S	S	S	S	S	S	S	S	S	S	S
9387	6	2013	Secretion	NR	-	Negative	**R**	S	S	S	S	S	S	S	S	S	S	I	S	S	S	S
9388	6	2013	Urine	NR	-	Negative	**R**	S	S	S	S	S	S	S	S	S	S	I	S	S	S	S
9925	7	2013	Abscess	NR	-	Negative	**R**	S	S	S	S	S	S	S	S	S	S	I	S	S	S	S
F2R9	NA	2003	Banana root	NR	-	Negative	**R**	S	S	S	S	S	S	S	S	S	S	S	S	S	S	S
T29A	NA	2003	Sugar cane stem	NR	-	Negative	**R**	S	S	S	S	S	S	S	S	S	S	S	S	S	S	S
6A2	NA	2003	Banana leaves	NR	-	Negative	**R**	S	S	S	S	S	S	S	S	S	S	S	S	S	S	S
VI	NA	2003	Banana stem	B	-	Negative	**R**	S	S	S	S	S	S	S	S	S	S	S	S	S	S	S
3	NA	2003	Maize shoots	B	-	Negative	**R**	S	S	S	S	S	S	S	S	S	S	S	S	S	S	S
CFNE-2006	NA	2003	Rice roots	NR	-	Negative	**R**	S	S	S	S	S	S	S	S	S	S	S	S	S	S	S

^a^Hospitlas: 1, Hospital de Niño y del Adolesente Morelense (HNAM); 2, Hospital del Niño de Tabasco (HNT);; 3, Hospital Infantil de Mexico (HIM); 4, Centro Regional de Control de Enfermedades Infecciosas (CRCEI); 5, Clinica del ISSSTE-Morelos (CHM); 6, Hospital Civil de Guadalajara (HCG); 7, Hospital Manuel Gea Gonzalez (HMGG).

^b^R = Resistant; I = Intermediate; S = Susceptible, according to CLSI^15^.

*Abbreviations*: Amoxicillin, AMX; Cephalothin, CF; Cefoxitin, FOX; Cefuroxime, CXM; Piperaciline, PIP; Piperaciline/Tazobactam, TZP; Aztreonam, ATM; Cefotaxime, CTX; Ceftazidime, CAZ, Gentamicin, GM; Amikacin, AMK; Ticarcillin, TIC; Tetracycline, TET; Nalidixic Acid, NAL; Ciprofloxacine, CIP; Levofloxacin, LUX.

One relevant finding of this study is that *K. variicola* isolates could be both multidrug resistant and sensitive. Given that *K. variicola* clinical isolates were obtained from patients where *K. pneumoniae* could also be isolated. The assumption that *K. variicola* could coexist in the same patient with *K. pneumoniae* was recently determined [28]. This notion reveals the possibility of *K. variicola* being able to acquire resistance genes to β-lactam and/or other antibiotics from some other bacteria, as could be the case of the ESBL SHV-type and CTX-M-15 genes identified in this study. The first family has been described in *K. pneumoniae* isolates; in fact, the CTX-M family is the most prevalent both in Mexico [16] and worldwide [29]. Previous studies have reported that isolates from the KpIII phylogenetic group displayed resistance to ampicillin, carbenicillin, piperacillin, gentamicin, ceftazidime, and ceftriaxone [24]. The resistance to penicillin antibiotics is in agreement with the LEN-type β-lactamases identified in the phylogenetic group KpIII [30]. In the present study, all *K. variicola* isolates studied were at least amoxicillin resistant (Table 2), most likely due to a chromosomal LEN-type β-lactamase.

### *rpoB* analysis and fingerprinting of the *K. variicola* isolates identified

**Figure 4:**
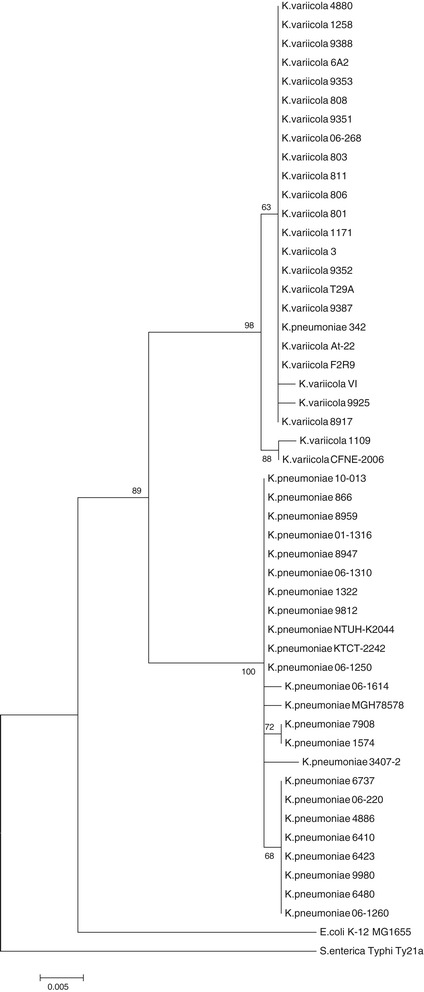
**The maximum-likelihood phylogeny of the** ***rpoB*** **sequences.** The tree was rooted with the sequences from *Escherichia coli* K-12 MG1655 and *Salmonella enterica* Ty21. To evaluate the support of the nodes, a bootstrap analysis of 100 replicates was conducted. For clarity, only the bootstrap values for the main groups are shown. The scale bar represents substitutions per site.

**Figure 5:**
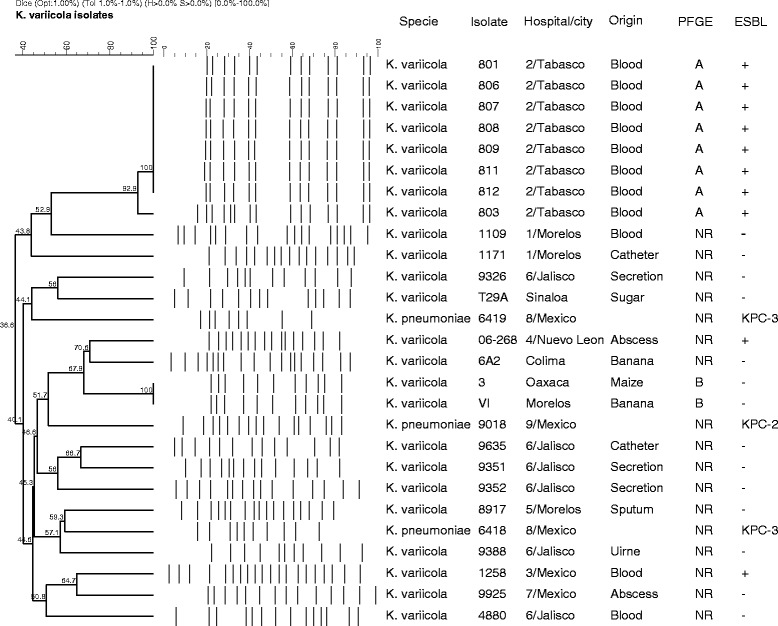
**PFGE and dendrogram analysis that includes** ***K. variicola*** **and** ***K. pneumoniae*** **isolates.** The figure shows the isolate (environmental and clinical), species, hospital and city, origin of sample, PFGE pattern and ESBL-producer phenotype.

The phylogeny of *rpoB* gene sequences of *K. variicola* and *K. pneumoniae* isolates clearly shows two strongly supported clusters (Figure 4); one corresponds to *K. variicola* isolates plus *K. pneumoniae* 342, whereas the other comprises *K. pneumoniae* isolates. In accordance with the approach (Figure 1), the phylogeny of *rpoB* sequences supports the differentiation of these related bacterial species. The relationship of *K. variicola* of clinical and environmental isolates was investigated by PFGE. The analysis showed two clonal groups (Figure 5 and Table 2). Clone A corresponds to *K. variicola* outbreak isolates from a pediatric hospital, whereas clone B cluster corresponds to two environmental isolates collected from maize and banana obtained from different cities (Figure 5). However, the fingerprinting analysis observed among *K. variicola* and *K. pneumoniae* isolates is not correlated with the bacterial species (Figure 5).

The multiplex PCR proposed in this study is an effective tool to differentiate *K. variicola* from *K. pneumoniae* because 100% of the clinical isolates were correctly identified, as were the control strains included. The reliability of the assay proposed was corroborated through the phylogenetic analysis of the *rpoB* gene from *K. variicola* and *K. pneumoniae* isolates identified previously by multiplex PCR. The sequence analysis of the *rpoB* gene was proven to be a useful tool for bacterial classification, particularly for *Enterobacteriaceae* species [31] and *K. variicola* [4]. The few shared unique genes to *K. variicola* and *K. pneumoniae* used in this study by multiplex PCR did not correspond to unique specific genes for each species. Instead, they corresponded to unique genes that clearly match with the *K. variicola* and *K. pneumoniae* genomes. The selected genes encode proteins that are involved in cellular metabolism in the case of *K. variicola*. We note that the shared unique genes of *K. variicola* analyzed by a recent BLASTn search have matches with a recently submitted *K. pneumoniae* KP5-1 genome, an isolate obtained from a known cotton boll pest. The housekeeping gene analysis of this *K. pneumoniae* KP5-1 isolate was compared with the housekeeping genes of *K. variicola* and *K. pneumoniae* genomes included in this study. The results showed that the *K. pneumoniae* KP5-1 genome fell within the *K. variicola* monophyletic group (Figure 1) and the chromosomal LEN-16 β-lactamase gene (Additional file 2). We considered that this bacterial genome corresponds to other *K. variicola* genomes deposited in GenBank database and is called a *K. pneumoniae* species.

## Conclusions

*K. variicola* and *K. pneumoniae* are closely related sister species that share many phenotypic properties; thus, *K. variicola* has been mistaken for *K. pneumoniae* for a long time and around the world. Our multiplex PCR provides the means to properly identify and genotype *K. variicola* and, therefore, this tool could be very helpful for clinical, epidemiological, population genetics and environmental studies of this species. For example, two recent reports have described *K. variicola* as a frequent cause of bloodstream infection, as being associated with higher mortality than *K. pneumoniae* [32], and as being associated with clinical bovine mastitis [33]. In addition, we determined that a previous collection of *K. pneumoniae* isolates has a low but significant prevalence of *K. variicola* isolates. We established that both multidrug-resistant and multidrug-sensitive isolates could colonize humans. We believe that our multiplex-PCR assay could be of paramount importance to understanding the population dynamics of *K. variicola* in both clinical and environmental settings.

## Additional files

Additional file 1:
**Clinical isolates of** ***K. pneumoniae*** **included in the study.**
Additional file 2:
**The maximum likelihood phylogeny of LEN-alleles amino acid sequences.** To evaluate the support of the nodes, a bootstrap analysis of 100 replicates was conducted and using the Jones-Taylor-Thornton substitution model. The scale bar represents substitutions per site. Additional file 3:
**Shared genes identified both** ***K. variicola*** **as** ***K. pneumoniae.***
Additional file 4:
**Metabolic and structural genes both** ***K. variicola*** **as** ***K. pneumoniae.***
