# Erratum to: Development of a Multiplex-PCR probe system for the proper identification of *Klebsiella variicola*

**DOI:** 10.1186/s12866-016-0652-4

**Published:** 2016-03-16

**Authors:** Ulises Garza-Ramos, Jesús Silva-Sánchez, Esperanza Martínez-Romero, Perla Tinoco, Marisol Pina-Gonzales, Humberto Barrios, Jesús Martínez-Barnetche, Rosa Elena Gómez-Barreto, Juan Tellez-Sosa

**Affiliations:** Departamento de Diagnóstico Epidemiológico, Av. Universidad # 655, Col. Sta. Ma. Ahuacatitlán, C.P. 62100 Cuernavaca, Morelos Mexico; Centro de Ciencias Genómicas (CCG), Universidad Nacional Autónoma de México (UNAM), Cuernavaca, Morelos Mexico; Instituto Nacional de Salud Pública (INSP), Centro de Investigación Sobre Enfermedades Infecciosas (CISEI), Departamento de Inmunología, Cuernavaca, México

## Erratum

The original version of this article [1] unfortunately contained a mistake in Table 1. The primer sequence which corresponds to KmtnC-R in the table 1 was provided incorrectly. The correct nucleotide sequence is:

KmtnC-R:GCATGGCCCAGGTGTTCAG

An updated version of Table 1 has been provided below.

**Table 1:** Amplification conditions, oligonucleotide combinations, sequence and amplification fragment of multiplex-PCR for *K. variicola* identification

Amplification conditions^a^	Name of combination primers	Shared unique genes, oligonucleotides and sequence (5′- 3′) of each bacterial specie					
		*K. pneumoniae*	Amplification fragment (bp)	*K. variicola*	Amplification fragment (bp)	*Klebsiella spp.*	Amplification fragment (bp)
		phosphohydrolase		phosphoglycerate mutase		phosphopentane phosphatase (mtnC)	
1	M-PCR-1	KP888-F: AAGCAAGCCAGAACAGAAAG	888	KV770-F: TCCCGAGGTTCACATTTCC	449	KmtnC-F: CCGCCGACCTTATCACTAC	340
		KP888-R: ACTTCGGTTTTATCCAGGTC		KV770-R: AGCGGGTGAACGTCGATAC		KmtnC-R: GCATGGCCCAGGTGTTCAG	
		transferase (*yphG*)		N-acetyltransferase		phosphopentane phosphatase (mtnC)	
1	M-PCR-2	KP878-F: ACCGATAACCAGCCTGACTT	878	KV1615-F: ACACAACATTTCAGGCGGCT	499	KmtnC-F: CCGCCGACCTTATCACTAC	340
		KP878-R: CTTTCTTCTGCCCACTGTTG		KV1615-R: GGGCGTGGCTTTTTTCATCG		KmtnC-R: GCATGGCCCAGGTGTTCAG	
		phosphohydrolase		thiopurine S-methyltransferase		phosphopentane phosphatase (mtnC)	
2	M-PCR-3	KP888-F: AAGCAAGCCAGA ACAGAAAG	888	KV1000-F: CTGGGATGTGGCAATGGTG	438	KmtnC-F: CCGCCGACCTTATCACTAC	340
		KP888-R: ACTTCGGTTTTATCCAGGTC		KV1000-F: AAACTGCGCCTGCTGTATC		KmtnC-R: GCATGGCCCAGGTGTTCAG	

^a^Multiplex-PCR conditions used under the oligonucleotides combinations. 1: 5pmol/reaction of *K. variicola* and *Klebsiella spp*, 25pmol/reaction of *K. pneumoniae*; 2: 25 pmol/reaction of *K. pneumoniae*, 5 pmol/reaction of *K. variicola* and 1 pmol/reaction of *Klebsiella spp*
