# Supplementary material for: Development of a Multiplex-PCR probe system for the proper identification of Klebsiella variicola
Source: BMC Microbiol. 2015 Mar 13;15:64. doi: 10.1186/s12866-015-0396-6 (PMC4361152; doi:10.1186/s12866-015-0396-6)
Supplement: Additional file 1: — Clinical isolates of K. pneumoniae included in the study. [file 12866_2015_396_MOESM1_ESM.docx]

Additional file 1. Clinical isolates of *K. pneumoniae* included in the study

| **Hospital** | **State** | **No. of isolates analized** | **No. of isolates obtained** | **Date of isolation** |
| --- | --- | --- | --- | --- |
| Hospital General de Campeche | Campeche | 6 | 11 | 2001- 2003 |
| Instituto de Ciencias Medicas y Nutrición, Salvador Zubirán | Distrito Federal | 9 | 20 | 1990, 1993, 1996- 2000, 2002 |
| Hospital Infantil CMN | Distrito Federal | 8 | 42 | 1991, 1996- 1997 |
| Hospital de Pediatría | Distrito Federal | 13 | 140 | 1996- 1997 |
| Hospital General de Acapulco | Guerrero | 25 | 25 | 2007- 2009,2012, 2013 |
| Hospital Civil de Guadalajara | Jalisco | 34 | 38 | 2006- 2009 |
| Hospital Infantil de Morelia, Eva Sámano de López Mateos | Michoacán | 16 | 67 | 1996, 1999- 2000 |
| Hospital General, José G. Parres | Morelos | 2 | 36 | 1996 |
| Hospital de Niño Morelense | Morelos | 3 | 3 | 2000- 2001, 2004 |
| Rio Mololoa | Nayarit | 5 | 5 |  |
| Hospital de altas especialidades No. 25, IMSS | Nuevo León | 4 | 4 | 2006- 2007 |
| Centro Regional de Control de Enfermedades Infecciosas | Nuevo León | 54 | 74 | 2006- 2008 |
| Hospital San José | Nuevo León | 1 | 1 | 2008 |
| Hospital Central, Dr. Ignacio Morones Prieto | San Luis Potosí | 7 | 19 | 2002- 2004 |
| Hospital Infantil de Sonora | Sonora | 4 | 4 | 2000- 2001 |
| ISSSTESON | Sonora | 1 | 1 | 2009 |
| Hospital del Niño de Tabasco | Tabasco | 1 | 6 | 1996 |
| Centro Médico de Especialidades, Dr. Rafael Lucio | Veracruz | 1 | 4 | 2008 |
| ISSSTE Centenario de la Revolución Emiliano Zapata | Morelos | 11 | 11 | 2011 |
| Hospital Infantil de México | Distrito Federal | 19 | 19 | 2011 |
| Hospital Civil de Guadalajara^a^ | Jalisco | 416 | 382 | 2006, 2009, 2011,2012,2013 |
| Hospital Monterrey | Nuevo León | 71 | 71 | 2011,2012 |
| Hospital Tabasco | Tabasco | 63 | 63 | 2011 |
| Hospital Regional Veracruz | Veracruz | 33 | 33 | 2011 |
| Hospital GEA Gonzalez^a^ | Distrito Federal | 221 | 221 | 2011,2012,2013 |
| Hospital Nayarit | Nayarit | 19 | 19 | 2011 |
| Hospital de Niño Morelense y del Adolocente | Morelos | 10 | 10 | 2011 |
| Hospital de Obregón | Sonora | 2 | 2 | 2012 |
| TOTAL | 12 | 1,060 | 1,331 |  |

^a^In these hospitals were included 557 clinical isolates susceptible to antibiotics: Hospital Civil de Guadalajara 352 isolates and Hospital GEA Gonzalez 151 isolates
