# Supplementary material for: Development of a Multiplex-PCR probe system for the proper identification of Klebsiella variicola
Source: BMC Microbiol. 2015 Mar 13;15:64. doi: 10.1186/s12866-015-0396-6 (PMC4361152; doi:10.1186/s12866-015-0396-6)
Supplement: Additional file 2: — The maximum likelihood phylogeny of LEN-alleles amino acid sequences. To evaluate the support of the nodes, a bootstrap analysis of 100 replicates was conducted and using the Jones-Taylor-Thornton substitution model. The scale bar represents substitutions per site. [file 12866_2015_396_MOESM2_ESM.docx]

Additional file 2. The maximum likelihood phylogeny of LEN-alleles amino acid sequences. To evaluate the support of the nodes, a bootstrap analysis of 100 replicates was conducted and using the Jones-Taylor-Thornton substitution model. The scale bar represents substitutions per site.
