# Supplementary material for: Development of a Multiplex-PCR probe system for the proper identification of Klebsiella variicola
Source: BMC Microbiol. 2015 Mar 13;15:64. doi: 10.1186/s12866-015-0396-6 (PMC4361152; doi:10.1186/s12866-015-0396-6)
Supplement: Additional file 3: — Shared genes identified both K. variicola as K. pneumoniae. [file 12866_2015_396_MOESM3_ESM.docx]

Additional file 3. Shared genes identified both *K. variicola* as *K. pneumoniae*

| **Functional category** | ***K. variicola*** | ***K. pneumoniae*** |
| --- | --- | --- |
| Hypothetical | 15 | 26 |
| Hypothetic conserved | 18 | - |
| Metabolic | 20 | 4 |
| Structural | 12 | 6 |
| Regulatory | 10 | 4 |
| Horizontal transfer | 3 | - |
| Other microorganisms | 36 | 14 |
| Total | 114 | 54 |
