# Supplementary material for: Development of a Multiplex-PCR probe system for the proper identification of Klebsiella variicola
Source: BMC Microbiol. 2015 Mar 13;15:64. doi: 10.1186/s12866-015-0396-6 (PMC4361152; doi:10.1186/s12866-015-0396-6)
Supplement: Additional file 4: — Metabolic and structural genes both K. variicola as K. pneumoniae. [file 12866_2015_396_MOESM4_ESM.docx]

Additional file 4. Metabolic and structural genes both *K. variicola* as *K. pneumoniae*

| ***K. variicola*** | |  | ***K. pneumoniae*** | |
| --- | --- | --- | --- | --- |
| **Metabolic proteins** | **Structural proteins** |  | **Metabolic proteins** | **Structural proteins** |
| Sugar transporter (SotB) | OsmC |  | Phosphohydrolase (domain HD) | Permease (maltose) |
| NAD-dependent epimerase | Membrane protein (putative) |  | Protein precursor of protein 1 (sulfatase) | Transporter protein (putative MFS) |
| Acetyl transferase family (GNATs) | Membrane integral protein |  | Homoserine dehydrogenase | Protein internal membranal (putative) |
| Glioxalasa | Membrane protein (putative) |  | Transferase (putative) | Protein internal membranal (putative) |
| Phosphoglycerate mutase (PGM) | Membrane protein (putative) |  |  | ATPase transporter of Mg+2 (putative) |
| nitrile hydratase (alpha subunit) | MipA |  |  | Amino-acid transporter |
| nitrile hydratase (beta subunit) | Periplasmic protein |  |  |  |
| Thiopurine S-methyltransferase | ABC peptide transporter |  |  |  |
| N-acetyltransferase | ABC sugar transporter (putative) |  |  |  |
| cysteine desulfurase (SufS Family) | ABC amino acid transporter |  |  |  |
| Adenyltransferase (putative) | Fimbrial protein |  |  |  |
| Cysteine synthase B (putative) | Protein with transmembranal domain |  |  |  |
| NifY | Protein with transmembranal domain |  |  |  |
| NifZ |  |  |  |  |
| NifM |  |  |  |  |
| NifQ |  |  |  |  |
| Gluconolactonase SMP-30 (LRE family) |  |  |  |  |
| Oxidereductase (FAD) |  |  |  |  |
| Proline racemase |  |  |  |  |
